# Texture Feature-Based Classification on Transrectal Ultrasound Image for Prostatic Cancer Detection

**DOI:** 10.1155/2020/7359375

**Published:** 2020-10-06

**Authors:** Xiaofu Huang, Ming Chen, Peizhong Liu, Yongzhao Du

**Affiliations:** ^1^College of Engineering, Huaqiao University, Quanzhou 362021, China; ^2^Zhangzhou Affiliated Hospital of Fujian Medical University, Zhangzhou 363000, China; ^3^Collaborative Innovation Center for Application Technology of Maternal and Infant Health Services, Quanzhou Medical College, Quanzhou 362022, China

## Abstract

Prostate cancer is one of the most common cancers in men. Early detection of prostate cancer is the key to successful treatment. Ultrasound imaging is one of the most suitable methods for the early detection of prostate cancer. Although ultrasound images can show cancer lesions, subjective interpretation is not accurate. Therefore, this paper proposes a transrectal ultrasound image analysis method, aiming at characterizing prostate tissue through image processing to evaluate the possibility of malignant tumours. Firstly, the input image is preprocessed by optical density conversion. Then, local binarization and Gaussian Markov random fields are used to extract texture features, and the linear combination is performed. Finally, the fused texture features are provided to SVM classifier for classification. The method has been applied to data set of 342 transrectal ultrasound images obtained from hospitals with an accuracy of 70.93%, sensitivity of 70.00%, and specificity of 71.74%. The experimental results show that it is possible to distinguish cancerous tissues from noncancerous tissues to some extent.

## 1. Introduction

Prostate cancer is one of the most common malignant tumours in the male genitourinary system. In recent years, its incidence and mortality rate in China has been increasing. In 2018, the China Cancer Center released the latest issue of national cancer statistics, pointing out that prostate cancer ranked sixth in the incidence rate of men in China, only lower than lung cancer, gastric cancer, liver cancer, esophageal cancer, and intestinal cancer [1]. The following year, the American Cancer Society released a data analysis report, showing that prostate cancer was the first in male morbidity and the second in deaths [2]. Prostate cancer has posed a serious threat to men's health, so early detection is particularly important.

At present, digital rectal examination, prostate-specific antigen, nuclear magnetic resonance imaging, and transrectal ultrasound are commonly used methods to examine prostate cancer [3]. Among them, a digital rectal examination is the most common and cheapest method to examine prostate cancer. However, the digital rectal examination cannot reach tumours in the anterior part of the prostate, which is easy to miss diagnosis [3]. Prostate-specific antigen concentration is a sensitive indicator for the diagnosis of prostate cancer, but some patients with benign prostate diseases will also have increased prostate-specific antigen concentration [4]. Therefore, prostate-specific antigen examination is easy to cause overdiagnosis, leading to unnecessary biopsy and potential overtreatment [5]. Nuclear magnetic resonance imaging (MRI) is an important examination technique for noninvasive evaluation of the prostate and its surrounding tissues, which has a relatively high diagnostic accuracy for prostate. Some studies have shown that compared with transrectal ultrasound-guided prostate biopsy, MRI-guided prostate biopsy can puncture targeted nodules with higher accuracy [6]. However, because MRI-guided biopsy requires special equipment, which is time-consuming and expensive, it cannot be popularized at present.

Transrectal ultrasound is generally used to guide prostate biopsy because of its visualization of prostate, nondamage, low cost, and real-time characteristics. A transrectal ultrasound-guided prostate biopsy is the gold standard for diagnosing prostate cancer. Although transrectal ultrasound is currently the most widely used imaging method, unfortunately, the visual interpretation of transrectal ultrasound images is poor and is not very reliable in distinguishing prostate cancer from normal glandular tissue. The diagnosis process will inevitably take the form of a tissue biopsy. However, a transrectal ultrasound-guided biopsy is to uniformly sample glands, not prostate cancer [7], and its positive diagnostic rate is shallow, especially for early prostate cancer lesions [8]. To obtain reliable results in histological analysis, multiple puncture biopsies are often required [9]. However, the increase of puncture times will bring a lot of pain to the patient (the probability of complications such as postoperative infection, hematuria, hematochezia, and the like will increase). At the same time, more clinically meaningless cancers will also be detected, resulting in excessive diagnosis and treatment [10].

Although the predictive value of positive results of transrectal ultrasound examination is very low (even trained urologists can hardly detect prostate cancer from ultrasound images), it is currently the most commonly used image detection method for diagnosing prostate cancer [3]. Improving the detection accuracy of transrectal ultrasound is helpful to reduce the number of puncture biopsies. Therefore, one possible way to improve the transrectal ultrasound-guided prostate puncture is to use computer-assisted analysis of transrectal ultrasound images.

Due to speckle noise, artifacts, attenuation, and signal loss inherent in transrectal ultrasound images, it is difficult for ultrasound doctors to analyze the image from the texture level to determine whether the image is positive (suffering from prostate cancer) or negative (normal). Therefore, this study uses a texture feature analysis method to try to obtain useful information from transrectal ultrasound images so as to improve the accuracy of prostate cancer detection.

We realize that our research is very difficult because specific pixels are not correctly marked. Ultrasound doctors are unable to analyze images at the microtexture level to determine whether pixels are positive or negative, and histological analysis of extracted tissues cannot be converted into pixel marker maps. Therefore, we can only use an imperfect label for all pixels in the biopsy area. Despite this problem, we have achieved some results, showing that it is possible to distinguish cancer tissues from noncancer tissues to a certain extent.

## 2. Related Work

Texture features consider the distribution of pixel intensity and the relationship between adjacent pixels [11, 12]. Different texture measurements often describe the corresponding texture from different angles. In medical imaging, since the internal structure of lesions can be quantitatively described, each texture feature is considered as an important indication feature for image pattern recognition [13, 14]. Previous studies have shown that heterogeneity reflected by texture features can be used to identify the nature of lesions with high diagnostic accuracy [15, 16].

In recent years, with the vigorous development of artificial intelligence, the technology of machine science has become increasingly mature. Many diagnostic methods based on computer-aided diagnosis provide convenience for medical diagnosis. In the field of vision research, medical image research mostly trains classic machine learning separators (such as support vector machines and decision trees) to extract human engineering-based features (such as texture and shape). So far, these algorithms have been successful applied to various medical applications such as liver [17], thyroid [18], and bladder cancer [19, 20]. However, although early detection of prostate cancer is very important, there is still little research on computer-aided detection of prostate cancer. Moreover, due to the number of patients used and the experimental techniques adopted, many of them are limited in scope, or the results cannot be considered representative. Llobet et al. [3] proposed a method of transrectal ultrasound image analysis for computer-aided diagnosis of prostate cancer. The best classification result of this research method reached a 61.6% area under the ROC curve. However, the recognition ability of urologists using the computer-aided system is only slightly improved compared with that of experts who do not use the system. Huynen et al. [21] developed a system for automatic analysis and interpretation of prostate ultrasound images. The system extracts five parameters of the cooccurrence matrix from ultrasound images to classify benign and cancerous prostate tissues. The sensitivity and specificity of this method are 80% and 88%, respectively, with good results. Kratzik et al. [22] published a study on prostate testing. The study used texture feature analysis to obtain good results (specificity and sensitivity both exceed 80%) but did not specify how to evaluate. Han et al. [23] proposed a prostate cancer detection method, which uses multiresolution autocorrelation texture features and clinical features. The method can effectively detect cancer tissues with a specificity of about 90% and a sensitivity of about 92%. However, this method is only applicable to similar databases. If other database data are used, such high sensitivity and specificity may not be achieved. Glotsos et al. [24] established a computer-aided diagnosis system based on texture analysis of transrectal ultrasound images. The system extracts 23 texture features from the region of interest in each image and uses exhaustive search (combining up to 5 features) and omission method to select and train the features of the classifier. In terms of overall system performance, the best classification accuracy rates for identifying normal, infectious, and cancer cases are 89.5%, 79.6%, and 82.7%. However, this research method is only suitable for use when data are scarce. Gomez-Ferrer and Arlandis [25] recorded 288 cases of transrectal ultrasound-guided biopsy and extracted three still images from the biopsy area. The texture features of ultrasonic images are obtained by “simple mapping” on grey and spatial grey correlation matrices. Finally, two methods based on nearest neighbour and Markov hidden model are used for classification. The nearest neighbour of the ROC curve is 59.7%, and the classification of Markov hidden model is 61.6%; ROC curve area of cooccurrence matrix is 60.1% nearest neighbour, and Markov hidden model is 60.0%. To solve the problems of unclear prostate boundary and insufficient data, Zhu et al. [26] proposed a boundary-weighted domain adaptive neural network (BOWDA-Net).

## 3. Materials and Methods

### 3.1. Data Procurement

**Figure 1:**
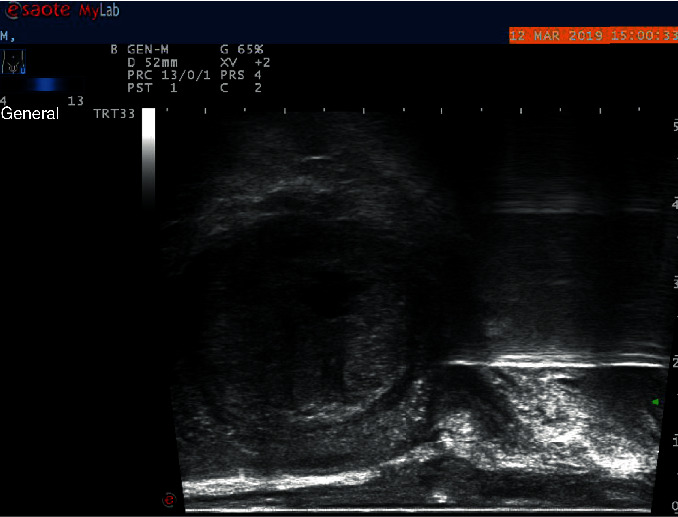
Biopsy tissue was recorded before the examination. In the image, the needle track is visible but has not been inserted into the prostate. Tissue and corresponding ultrasonic texture are not disturbed, and this image is used for image processing and texture analysis.

**Figure 2:**
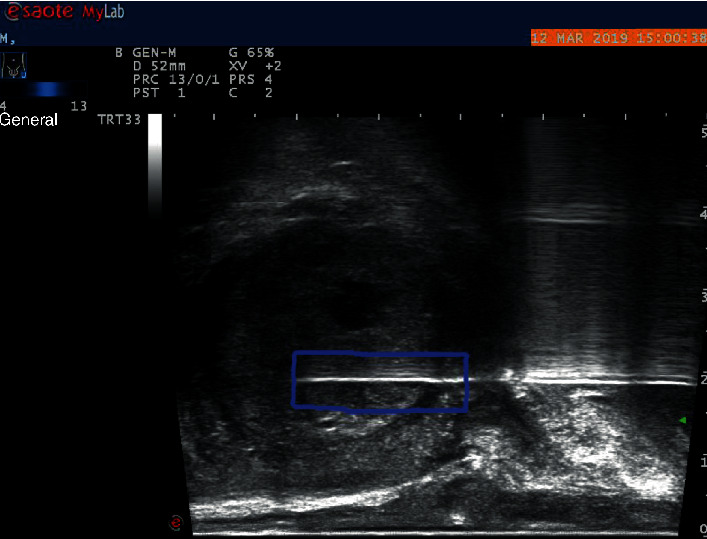
Biopsy needle track can still be seen in the prostate gland. In this picture, the puncture position is determined. The extracted tissue is analyzed by a pathologist, and the puncture position determines the analysis position in a clean image (Figure 1).

In Zhangzhou Hospital, a transrectal ultrasound-guided prostate biopsy is usually performed for all patients with prostate cancer-related symptoms (such as high PSA value and abnormal DRE results). The inserted transrectal ultrasound probe displays sagittal images of the prostate. When suspicious areas are found, the biopsy needle connected to the probe will be triggered for tissue extraction and later histological analysis. Generally, multiple biopsies will be performed if no particularly suspicious area is found. According to the guidance of ultrasound doctors, our experimental data are mainly pictures before biopsy (Figure 1). Because histology can only be determined from the resected tissue, the puncture location must be accurately known. To achieve this, we used the second image, which was recorded before the biopsy needle was retracted from the gland (Figure 2). There is a white needle track in each image. In each biopsy, we define a point for the first needle where the probe appears in front of the prostate and define a second point for the position where the probe is inserted into the prostate about two scales from the first point. We define a rectangle based on these two points (as shown in Figure 2). Since there is no obvious patient movement during the biopsy, pixels in the previous image are marked with the defined rectangle. The image of Figure 1 is our experimental data. In Figure 1, we manually cut a rectangular image at the same position as that of Figure 2, which is the region of interest for our experiment.

Histological analysis can indicate whether the extracted tissue has prostate cancer, and if so, its location can also be known. However, in a clinical environment, it is difficult to carry out reliable physical labelling on the extracted tissue and then map the physical labelling to pixel labelling. Therefore, we will use a label definition for pixels in all biopsy areas, which means that some images marked as positive samples may contain normal tissues. Fortunately, however, an image pixel marked negative always corresponds to normal tissue, because histological examination can ensure that the entire biopsy area is free of prostate cancer.

### 3.2. Method

**Figure 3:**
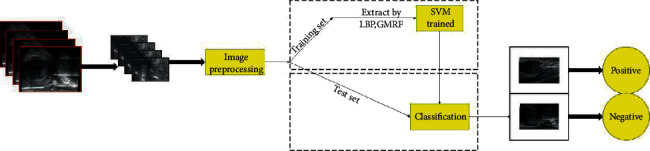
Method flow chart.

Figure 3 shows the flow of the proposed classification method. The whole method flow mainly includes four parts: image preprocessing, feature extraction, feature fusion, and classification. The main contribution lies in the use of optical density conversion technology to increase the contrast of the image and reduce its noise. Gaussian Markov random field and local binarization are used to extract the two texture features of the image, and then the two features are linearly combined. Finally, the fused features are put into SVM classifier for classification.

#### 3.2.1. Optical Density Image Processing Technology

**Figure 4:**
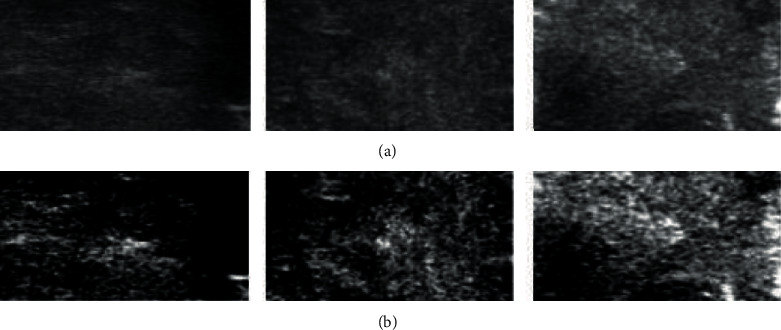
(a) Transrectal ultrasound prostate image and (b) optical density image.

Removing noise from original images is still a challenging research topic in image processing. Generally speaking, there is no commonly used noise reduction enhancement method. Usually, the appropriate noise reduction method is selected according to the image characteristics. Limited by the principle of ultrasonic imaging and the hardware itself, the quality of ultrasonic images is not satisfactory. The main manifestations are of low contrast and speckle noise. Therefore, this paper uses an optical density conversion technology [27] to dry the selected region of interest and enhance the contrast and to make the details of the image clearer and more obvious, which is conducive to the subsequent analysis and processing of the image. The optical density transformation for each pixel (*i*, *j*)of an object region is defined as follows:

(1) $$\begin{array}{c} {\text{OD}}_{ij}=\log\left(\frac{I_{ij}}{I_{o}}\right), \end{array}$$

where *I*_ij_ is the intensity value of pixel, and *I*_*o*_ is the average intensity. In this method, the intensity of gray image is converted into optical density, and each optical density value is linearly mapped to the image with 8-bit depth information, so that the optical density image can be obtained. As shown in Figure 4.

#### 3.2.2. Feature Extraction

Texture features can reflect the overall change of grey pixel values in the image, and different tissues have different textures. Therefore, by distinguishing and identifying texture features in transrectal ultrasound prostate images, suspected case samples with similar texture structures to confirmed case samples can be detected, thus providing decision support for doctors. As one of the most widely used and basic image global features, there are many texture feature extraction methods, which are often used in medical ultrasound image analysis: grey level cooccurrence matrices (GLCM) [28], histogram of oriented gradient (HOG) [29], local binary pattern (LBP) [30], etc. Each method has its advantages and disadvantages. In actual use, it is often necessary to select the corresponding feature extraction method according to the practical application requirements.

According to the relevant research results in recent years, different types of texture features are generally complementary. In the image classification task, the combination of different feature extraction methods can often achieve higher classification accuracy than when used alone. Therefore, we use local binarization and Gaussian Markov random field model to extract texture features.

(1) LBP. LBP (local binary pattern) [30] is an operator used to describe local texture features of images. Its basic idea is defined in the eight fields of pixels (3x3 window). The grey value of the central pixel is taken as the threshold, and the values of the surrounding 8 pixels are compared with it. If the surrounding pixel value is less than the grey value of the central pixel, the pixel value is marked as 0; otherwise, it is marked as 1. In this way, 8 points in the domain size of 3x3 can be compared to generate 8-bit binary numbers (usually converted into a decimal, i.e., LBP code, 256 kinds in total). Each pixel obtains a binary combination, i.e., LBP value of pixel point in the centre of the window, and this value is used to reflect texture information of the region. However, as the image rotates, the pixels in the neighbourhood will recombine, and the LBP value will change. To keep LBP rotation unchanged, Ojala et al. [30] improved the LBP operator. The formula is as follows:
  (2) $$\begin{array}{c} \left\{\begin{array}{c} {\text{LBP}}_{N,R}\left(a,b\right)=\overset{N-1}{\underset{i=0}{\sum }}s\left(G_{i}-G_{0}\right)•2^{i}\\ s\left(t\right)=\left\{\begin{array}{c} 1,t\geq 0\\ 0,\text{else} \end{array}\right., \end{array}\right. \end{array}$$
  where *R* is the radius of the neighbourhood circle, and *N* is the number of pixels evenly distributed in the neighbourhood. *G*_*i*_ represents *N* pixels centred on *G*_0_.
(2) GMRF. Gaussian Markov random field (GMRF) model [31] is a probability model to describe the image structure and is a better method to describe the texture. It was originally described by Leonard E. Baum and other authors in a series of statistical papers in the second half of the 1960s. There is a certain correlation between the category of a pixel in an image and the category of pixels in its surrounding areas. This correlation is called Markov correlation. An image can be regarded as a two-dimensional random process, and the distribution of image data can be described by conditional probability. MRF assumes that the pixel value of each pixel in the image depends only on the pixel value of the pixel in its domain. A Markov random field is usually described by the following local conditional probability density (PDF):
  (3) $$\begin{array}{c} p\left(\left(m,n\right)\;|\;f\left(k,l\right),\left(k,l\right)\neq \left(m,n\right),\left(k,l\right)\in \wedge \right)=p\left(f\left(m,n\right)\;|\;f\left(k,l\right),\left(k,l\right)\in N_{\left(m,n\right)}\right). \end{array}$$
  *N*(*m*, *n*) is the neighbourhood pixel point of the centre pixel. If PDF follows Gaussian distribution, MRF is called GMRF. Its prominent feature is that it introduces structural information through a properly defined neighbourhood system and provides a model commonly used to express the interaction between spatially related random variables. The parameters generated from this model can describe the aggregation characteristics of textures in different directions and forms.

#### 3.2.3. Feature Fusion

Both LBP texture features and GMRF texture features have strong capabilities in feature extraction of transrectal ultrasound prostate images, but they have some limitations in practical application. In the process of feature extraction, LBP only considers the grey values of other surrounding pixels, but does not fully consider the interaction and interdependence between the central pixel and the surrounding pixels. These dependencies can be random, functional, or structural and can be represented by Gaussian Markov random field model. Therefore, this paper first extracts LBP features from transrectal ultrasound prostate images and then calculates the conditional probability density of the extracted LBP feature images.

#### 3.2.4. Classifier Design

Image classification is an important research field and has practical applications in many areas such as pattern recognition, artificial intelligence medicine, and visual analysis. For image classification, we adopt SVM classifier, which is described in detail as follows.

SVM is based on statistical learning technology and is the foundation of modern statistical learning theory. It was proposed by Cortes and Vapnik in 1995 [32]. SVM algorithm is a supervised machine learning algorithm by minimizing empirical errors and maximizing geometric edges to complete pattern classification and regression analysis. It is widely used in statistical classification and regression analysis. It has unique advantages in solving small samples and nonlinear high-dimensional pattern recognition and can be widely applied to machine learning problems such as function fitting. The basic principle of the modified method is to find the fractal hyperplane of the training set *n* in the sample space and to separate the categories to the maximum extent. Besides, SVM, as a quadratic programming problem, can find a globally unique optimal solution, thus avoiding the occurrence of local minima. The principle and solving process are as follows:

Given a data set:

(4) $$\begin{array}{c} N\left\{\left(x_{i},y_{i}\right)\;|\;x_{i}\in R^{n},y_{i}\in \left\{-1,+1\right\},i=1,\cdots n\right\}. \end{array}$$

Then, the discriminant function of SVM is as follows:

(5) $$\begin{array}{c} f\left(x\right)=\mathrm{sign}\left(\overset{n}{\underset{i=1}{\sum }}A_{i}y_{i}K\left(x,x_{i}\right)+B\right), \end{array}$$

where *K*(*x*, *x*_*i*_) is the kernel function, and *n* is the number of support vector machines. The kernel function is vital in support vector training. It can effectively overcome the dimension disaster problem. Proper kernel function can improve the prediction accuracy of the classification model. Common kernel functions include Gaussian function, polynomial function, sigmoid function, and linear function. In this paper, input vectors composed of texture features are selected as Gaussian functions. The classification results of SVM are used to distinguish positive samples from negative samples in transrectal ultrasound images.

In this paper, SVM classification data are using a linear hyperplane that separates data into two isolated classes. This hyperplane is calculated using Gaussian kernel function. The number of neighbours in k-nearest neighbour (KNN) [33] is set to 5. The confidence factor of decision tree (DT) [34] is set to 0.25, and the minimum case tree of each leaf is set to 2. Random forest (RF) [35] is using matlab random forest toolbox, with trees selection of 500 and mtry of 61.

## 4. Experimental Results

### 4.1. Experimental Data

This research has been approved and reviewed by the local ethics committee, and all relevant topics have been notified with permission. Transrectal ultrasound prostate images used in this experiment were from Zhangzhou Hospital affiliated to Fujian Medical University, with a total of 48 cases. All pathological cases were biopsied under ultrasound guidance by experienced pathologists and confirmed histologically. The data collection time is from March 2019 to November 2019, and each patient file contains multiple images. The data are classified according to pathological results. There were 36 cases in training set and 12 cases in test set. Experiments were conducted on prostate diagnosis to distinguish whether transrectal ultrasound images have prostate cancer. Therefore, the negative samples of training data were 18 cases (126 images), and the positive samples were 18 cases (130 images). The remaining 12 cases were used as experimental test sets, of which 6 cases (40 images) were positive samples, and 6 cases (46 images) were negative samples.

### 4.2. Experimental Setup and Performance Evaluation

The experiment is completed based on Windows10 operating system. The computer hardware is configured as follows: Intel(R) Core(TM) i7-8700 is used for CPU, NVIDIA GeForce GTX-1080Ti is used for GPU, and the video memory is 11G and the memory is 32G. The programming environment is Matlab2017a.

Disease classification results are true positive, true negative, false positive, and false negative. In order to facilitate comparative analysis with the existing methods, we have considered three indicators: accuracy (ACC), sensitivity (SEN), and specificity (SPEC) [36], as shown in Table 1. Among them, TP, TN, FP, and FN are the number of true positive, true negative, false positive, and false negative, respectively, in the classification results. Accuracy is a direct measure of comparison between methods. Sensitivity and specificity describe how diagnostic tests capture the real presence or absence of disease. These evaluation indexes together describe the accuracy and error rate of image classification and recognition methods. Among them, the higher the accuracy, sensitivity, and specificity, the lower the error rate of the method.

### 4.3. Comparison of Characteristic Combination Performance

In order to test the effectiveness of the combination of Gaussian Markov random field and local binarization, we respectively use a variety of texture features to carry out experiments and compare the accuracy with the proposed method. In all experiments, we use support vector machine to classify. The classification performance of different methods is shown in Table 2. All the values in Table 2 are obtained using our data set. As can be seen from the table, compared with individual features, the classification accuracy of feature fusion has been significantly improved, and other indicators have also been improved to varying degrees, especially the specificity indicators are more obvious. Compared with the classification results of different texture features in Table 2, the texture feature fusion classification results proposed in this paper are the best, with the classification accuracy rate reaching 70.93%, sensitivity 70.00%, and specificity 71.74%. Using Gaussian Markov random fields to extract texture features alone does not provide meaningful results for our data set. As can be seen from Table 2, our method has high specificity while maintaining high sensitivity.

### 4.4. Performance Comparison of the Methods

To test the effectiveness of our approach, we compared our method with the following three ways: (a) KNN classifier [33], (b) decision tree (DT) classifier [34], and (c) random forest (RF) classifier [35]. Specifically, in each experiment, the image is preprocessed by optical density conversion technology, and then the Gaussian Markov random field and local binarization features are extracted and fused. Finally, the above three classifiers are used for classification.

Compared with the classification results of different classifiers in Table 3, the classification results of support vector machine are higher than those of other classifiers, with the classification accuracy rate reaching 70.93%, sensitivity reaching 70.00%, and specificity reaching 71.74%. The second is DT, with a classification accuracy of 63.96%, sensitivity of 55%, and specificity of 71.72%. The classification accuracy of KNN was 63.95%, sensitivity 57.50%, and specificity 69.57%. The classification accuracy of RF was 62.78%, sensitivity 62.50%, and specificity 63.04%. As can be seen from the results shown in Table 3, our proposed method has better performance than other methods.

By comparing the experimental results in Tables 2 and 3, it can be found that LBP+GMRF+SVM proposed in this paper gives full play to the complementarity of texture features. The classification accuracy of this method is 4.65% higher than the highest accuracy of single feature. At the same time, the accuracy of this method is 6.97% higher than highest accuracy of other classifiers.

### 4.5. Effect Analysis of Image Preprocessing

The experimental data are preprocessed, and the texture features are analyzed by the SVM classifier. As shown in Table 4, preprocessing helps to extract more useful features from images and effectively improves classification accuracy.

### 4.6. Method Performance Evaluation

Aiming at the problem of small amount of data sets, 5-fold cross-validation is used to verify the effectiveness of the proposed method. That is, the whole data set is divided into five different subsets. Every time one subset is used as the test set and the other four subsets are used as the training set, this process is repeated five times. Finally, the average of five experimental results is calculated to evaluate the performance of the classifier.

By comparing Table 3 with Table 5, it can be found that the error between the results of 5-fold cross-validation and the experimental results of dividing the data set into training set and test set is not great. This verifies the effectiveness of the proposed method.

## 5. Discussion

Since TRUS cannot reliably identify prostate cancer [8], 6-18 or more puncture biopsies [9] are used to detect cancerous lesions. However, some biopsy samples taken from some male patients will not contain cancer. Also, clinically significant PSA does not necessarily have prostate cancer [4].

Prostate cancer is hypoechoic in ultrasound images [38]. Therefore, TRUS has poor visual interpretation and cannot accurately identify the tumour area. Gomez-Ferrer and Arlandis [25] found only 12.6% hypoechoic lesions in their work, which were found in most (68%) benign tissues. These data confirm the need to try to analyze transrectal ultrasound images with computer assistance. Therefore, this paper proposes an image analysis method based on texture feature fusion.

Sensitivity is an essential criterion for medical diagnosis, especially in the early stage of disease examination. Positive samples of clinical examination should avoid missed diagnosis as much as possible. The texture feature fusion method is used in this experiment. The sensitivity of the fused texture feature is 70.00%, which is better than 67.50% of LBP and 57.50% of GMRF. This shows that there is a correlation between texture description and sensitivity in the image: the more texture descriptions, the more obvious the features of positive lesions. However, for transrectal ultrasound images with small differences between classes, the overall classification performance will also decrease. Experiments show that texture feature fusion has a significant effect on the classification of transrectal ultrasound images.

According to our experimental methods and results, it is quite difficult to develop software for real-time image recognition in the future. Because images will have to be analyzed in real-time and suspicious areas identified, we believe that this may be due to several factors: the method or the disease itself. It may be that prostate cancer and its histological changes have different structures from normal glands. Another problem we are facing is incomplete labelling when conducting such studies because it is almost impossible to accurately determine the exact location of cancer in transrectal ultrasound images with current technologies. In our research, this annotation was obtained by studying histological analysis and puncture site location. However, this may be affected more or less because the length of the region of interest we extract rarely corresponds to cancer.

## 6. Conclusion

This paper proposes a texture feature analysis method to improve the classification accuracy of transrectal ultrasound prostate images. Firstly, the transrectal ultrasound image is preprocessed by optical density conversion technology, and then Gaussian Markov random fields and local binarization features are extracted. The two features are linearly combined, and then SVM classifier is used for classification experiments. Finally, several comparative experiments were carried out on the data set we collected, and the experimental results were given and analyzed. The experimental results show that the method has good classification accuracy (70.93%), sensitivity (70%), and specificity (71.74%). This provides a low cost, rapid, and repeatable analysis method for transrectal ultrasound-guided prostate puncture. In the future work, we plan to carry out more effective cooperation with hospitals to obtain more data sets, and then we will improve the proposed method to make it more suitable for the actual needs of the medical field.

## Figures and Tables

**Table 1:** Definition of evaluation index.

Evaluations	Definition
ACC	(TP + TN)/(TP + TN + FP + FN)
SEN	TP/(TP + FN)
SPEC	TN/(TN + FP)

**Table 2:** Classification accuracy with different types of features.

Method	ACC	SEN	SPEC
GLCM [28]	61.63%	67.50%	56.52%
HOG [29]	66.28%	65.00%	67.39%
LBP [30]	60.47%	67.50%	54.35%
GMRF [31]	53.49%	57.50%	50.00%
GLDS [37]	61.63%	62.50%	60.87%
Our method	70.93%	70.00%	71.74%

**Table 3:** Classification performance of all comparison methods.

Method	ACC	SEN	SPEC
KNN [33]	63.95%	57.50%	69.57%
DT [34]	63.96%	55.00%	71.72%
RF [35]	62.78%	62.50%	63.04%
Our method	70.93%	70.00%	71.74%

**Table 4:** The classification accuracy (%) based on transrectal ultrasound image preprocessing.

ACC (%)	LBP	HOG	GMRF	GLDS	GLCM	LBP+GMRF
Before preprocessing	55.81	58.14	50.00	60.47	53.49	58.14
After preprocessing	60.47	66.28	53.49	61.63	61.63	70.93

**Table 5:** Average test results of 5-fold cross-validation.

Method	ACC	SEN	SPEC
KNN [33]	62.73%	58.84%	66.81%
DT [34]	60.83%	56.30%	64.99%
RF [35]	64.04%	66.47%	65.14%
SVM [32]	70.11%	68.26%	71.97%

## Data Availability

The (transrectal ultrasound images) data used to support the findings of this study were supplied by Zhangzhou Affiliated Hospital of Fujian Medical University, China, under license and so cannot be made freely available.
